# Directional Switching Mechanism of the Bacterial Flagellar Motor

**DOI:** 10.1016/j.csbj.2019.07.020

**Published:** 2019-07-31

**Authors:** Tohru Minamino, Miki Kinoshita, Keiichi Namba

**Affiliations:** a Graduate School of Frontier Biosciences, Osaka University, 1-3 Yamadoaka, Suita, Osaka 565-0871, Japan; b RIKEN Center for Biosystems Dynamic Research & Spring-8 Center, 1-3 Yamadaoka, Suita, Osaka 565-0871, Japan

**Keywords:** Adaptive remodeling, Bacterial flagellar motor, Chemotaxis, Cooperativity, Directional switching, Motility

## Abstract

Bacteria sense temporal changes in extracellular stimuli via sensory signal transducers and move by rotating flagella towards into a favorable environment for their survival. Each flagellum is a supramolecular motility machine consisting of a bi-directional rotary motor, a universal joint and a helical propeller. The signal transducers transmit environmental signals to the flagellar motor through a cytoplasmic chemotactic signaling pathway. The flagellar motor is composed of a rotor and multiple stator units, each of which acts as a transmembrane proton channel to conduct protons and exert force on the rotor. FliG, FliM and FliN form the C ring on the cytoplasmic face of the basal body MS ring made of the transmembrane protein FliF and act as the rotor. The C ring also serves as a switching device that enables the motor to spin in both counterclockwise (CCW) and clockwise (CW) directions. The phosphorylated form of the chemotactic signaling protein CheY binds to FliM and FliN to induce conformational changes of the C ring responsible for switching the direction of flagellar motor rotation from CCW to CW. In this mini-review, we will describe current understanding of the switching mechanism of the bacterial flagellar motor.

## 1 Introduction

**Fig. 1:**
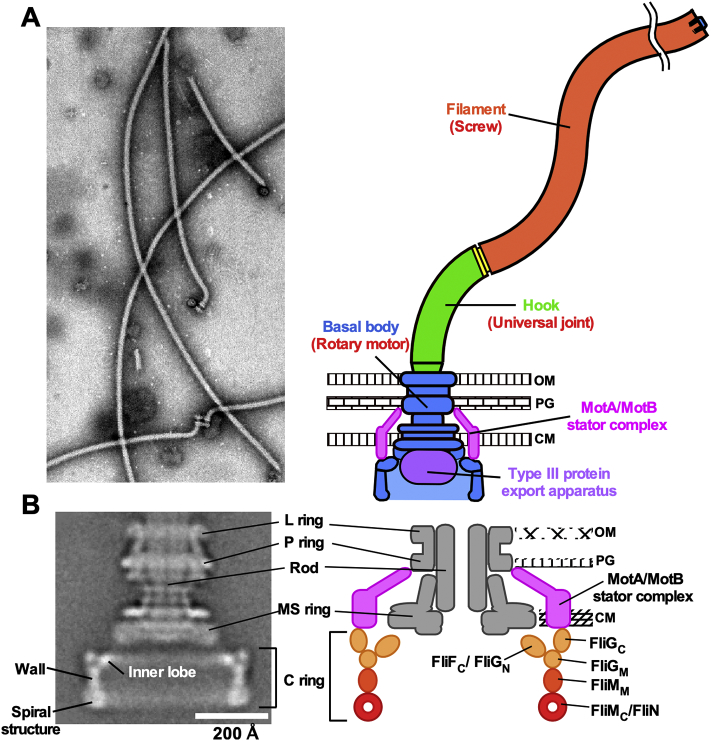
Subunit organization in the flagellar motor. (A) Bacterial flagella. Electron micrograph of flagella purified from *Salmonella* on the left and its schematic diagram on the right. The flagellum is composed of the bsal body as a rotary motor, the hook as a universal joint and the filament as a molecular screw. (B) CryoEM image of *Salmonella* basal body on the left and its schematic diagram on the right. The purified basal body consists of the C, MS, L and P rings and the rod. A dozen MotA/MotB stator complexes are associated with the basal body but are lost during purification. The C ring is composed of FliG, FliM and FliN. The N-terminal domain of FliG (FliG_N_) forms the inner lobe along with the C-terminal cytoplasmic domain of FliF (FliF_C_). The C-terminal domain of FliG (FliG_C_) is located in the upper part of the C ring wall. The middle domain of FliM (FliM_M_) is located between the middle domain of FliG (FliG_M_) and FliN and forms a cylindrical wall of the C ring. A continuous spiral density at the bottom edge of the C ring is made of the C-terminal domains of FliM (FliM_C_) and FliN.

Many bacteria possess flagella to swim in liquid media and move on solid surfaces. *Escherichia coli* and *Salmonella enterica* serovar Typhimurium (hereafter referred to *Salmonella*) are model organisms that have provided deep insights into the structure and function of the bacterial flagellum. The flagellum is composed of basal body rings and an axial structure consisting of at least three parts: the rod as a drive shaft, the hook as a universal joint and the filament as a helical propeller (Fig. 1A). The flagellar motor of *E. coli* and *Salmonella* consists of a rotor and a dozen stator units and is powered by an electrochemical potential of protons across the cytoplasmic membrane, namely proton motive force. Marine *Vibrio* and extremely alkalophilic *Bacillus* utilize sodium motive force as the energy source to drive flagellar motor rotation. The rotor is composed of the MS ring made of the transmembrane protein FliF and the C ring consisting of three cytoplasmic proteins, FliG, FliM and FliN. Each stator unit is composed of two transmembrane proteins, MotA and MotB, and acts as a transmembrane proton channel to couple the proton flow through the channel with torque generation (Fig. 1B) [[1], [2], [3], [4], [5]].

The flagellar motor rotates in either counterclockwise (CCW; viewed from the flagellar filament to the motor) or clockwise (CW) direction in *E. coli* and *Salmonella*. When all the motors rotate in the CCW direction, flagellar filaments together form a bundle behind the cell body to push the cell forward. Brief CW rotation of one or more flagellar motors disrupts the flagellar bundle, allowing the cell to tumble, followed by a change in the swimming direction. Sensory signal transducers sense temporal changes in extracellular stimuli such as chemicals, temperature and pH and transmit such extracellular signals to the flagellar motor via the intracellular chemotactic signaling network. The phosphorylated form of CheY (CheY-P), which serves as a signaling molecule, binds to FliM and FliN in the C ring to switch the direction of flagellar motor rotation from CCW to CW. Thus, the C ring acts as a switching device to switch between the CCW and CW states of the motor [2,5].

The stator complex is composed of four copies of MotA and two copies of MotB. The MotA_4_/MotB_2_ complex is anchored to the peptidoglycan (PG) layer through direct interactions of the C-terminal periplasmic domain of MotB with the PG layer to become an active stator unit around the rotor [4]. A highly conserved aspartate residue of MotB (Asp-32 in the *E. coli* protein and Asp-33 in the *Salmonella* protein) is located in the MotA_4_/MotB_2_ proton channel and is involved in the energy coupling mechanism [6,7]. The cytoplasmic loop between transmembrane helices 2 and 3 of MotA (MotA_C_) contains highly conserved Arg-90 and Glu-98 residues and are important not only for torque generation but also for stator assembly around the rotor [[8], [9], [10]].

FliG is directly involved in torque generation [8]. Highly conserved Arg-281 and Asp-289 residues are located on the torque helix of FliG (Helix_Torque_) [11] and interact with Glu-98 and Arg-90 of MotA_C_, respectively [8,10]. Since the elementary process of torque generation caused by sequential stator–rotor interactions in the flagellar motor is symmetric in the CCW and CW rotation, Helix_Torque_ is postulated to rotate 180° relative to MotA_C_ in a highly cooperative manner when the motor switches between the CCW and CW states of the C ring [12]. This mini-review article covers current understanding of how such a cooperative remodeling of the C ring structure occurs.

## 2 Structure of the C Ring

**Fig. 2:**
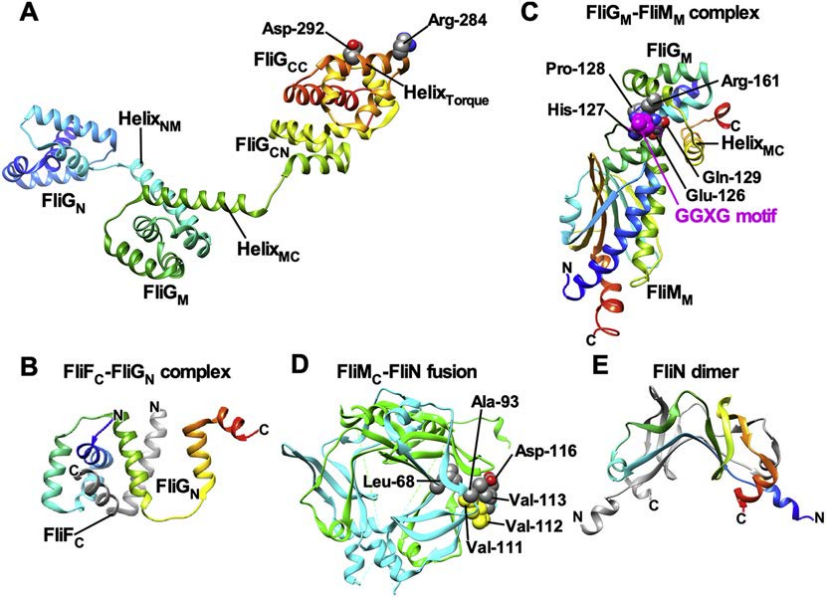
Crystal structures of C ring proteins. (A) Crystal structure of FliG derived from *Aquifex aeolicus* (PDB code: 3HJL). The Cα backbone is colour-coded from blue to red, going through the rainbow colors from the N- to the C-terminus. FliG consists of FliG_N_, FliG_M_ and FliG_C_ domains and two helix linkers, Helix_NM_ and Helix_MC_. FliG_C_ is divided into FliG_CN_ and FliG_CC_ subdomains. Arg-284 and Asp-292 residues, which correspond to Arg-281 and Asp-289 of *E. coli* FliG, respectively, are located in the torque helix of FliG_CC_ (Helix_Torque_), which is involved in electrostatic interactions with the cytoplasmic loop of MotA. (B) Crystal structure of the FliF_C_/FliG_N_ complex derived from *Thermotaoga maritima* (PDB code: 5TDY). FliF_C_ consisting of two α-helices (grey) binds to a hydrophobic groove of FliG_N_ (rainbow). (C) Crystal structure of the FliG_M_/FliM_M_ complex derived from *T. maritima* (PDB code: 3SOH). A well conserved EHPQR motif in FliG_M_ and a well conserved GGXG motif in FliM_M_ are responsible for the FliG_M_–FliM_M_ interaction. (D) Crystal structure of *Salmonella* FliM_C_-FliN_N_ fusion protein (PDB code: 4YXB). FliM_C_ and FliN_N_ subunits are shown in green and cyan, respectively. Leu-68, Ala-93, Val-113 and Asp-116 of FliN are involved in the interaction with CheY-P. Val-111, Val-112 and Val-113 of FliN are required for the interaction with FliH. (E) Crystal structure of the FliN dimer derived from *T. maritima* (PDB code: 1YAB). (For interpretation of the references to colour in this figure legend, the reader is referred to the web version of this article.)

FliF assembles into the MS ring within the cytoplasmic membrane [13]. The C ring consisting of a cylindrical wall and inner lobes is formed by FliG, FliM and FliN on the cytoplasmic face of the MS ring with the inner lobes connected to the MS ring (Fig. 1B) [14]. FliF requires FliG to facilitate MS ring formation in the cytoplasmic membrane [15]. FliG binds to FliF with a one-to-one stoichiometry [16]. FliM and FliN together form the FliM_1_/FliN_3_ complex consisting of one copy of FliM and three copies of FliN [17], and the FliM_1_/FliN_3_ complex binds to the FliG ring structure through a one-to-one interaction between FliG and FliM to form the continuous C ring wall [[18], [19], [20]]. Most of the domain structures of FliG, FliM and FliN have been solved at atomic resolution (Fig. 2), and possible models of their organization in the C ring have been proposed (Fig. 1B) [21,22].

### 2.1 FliG

FliG consists of three domains: N-terminal (FliG_N_), middle (FliG_M_) and C-terminal (FliG_C_) domains (Fig. 2A) [23]. FliG_C_ is divided into two subdomains: FliG_CN_ and FliG_CC_. FliG_N_ is involved in the interaction with the C-terminal cytoplasmic domain of FliF (FliF_C_) (Fig. 2B) [24,25]. Inter-molecular interactions between FliG_N_ and FliG_N_ and between FliG_M_ and FliG_CN_ are responsible for the assembly of FliG into the ring structure on the cytoplasmic face of the MS ring [[26], [27], [28], [29]]. FliG_M_ provides binding sites for FliM (Fig. 2C) [[18], [19], [20]]. A highly conserved EHPQR motif of FliG_M_ is involved in the interaction with FliM [18,30]. FliG_CC_ contains Helix_Torque_, and highly conserved Arg-284 and Asp-292 residues of *Aquifex aeolicus* FliG, which corresponds to Arg-281 and Asp-289 of *E. coli* FliG involved in the interactions with conserved charged residues of MotA_C_ [8,11], are exposed to solvent on the surface of Helix_Torque_ [23].

### 2.2 FliM

FliM consists of three domains: N-terminal (FliM_N_), middle (FliM_M_) and C-terminal (FliM_C_) domains [31,32]. FliM_N_ contains a well conserved LSQXEIDALL sequence, which is responsible for the interaction with CheY-P [33]. FliM_N_ is intrinsically disordered, and the binding of CheY-P to FliM_N_ allows FliM_N_ to become structured [32]. FliM_M_ has a compactly folded conformation (Fig. 2C), and side-by-side associations between FliM_M_ domains are responsible for the formation of the C ring wall [32]. The binding of CheY-P to FliM_N_ affects inter-molecular FliM_M_–FliM_M_ interactions, thereby inducing a conformational change in the C ring responsible for switching the direction of flagellar motor rotation [34]. A well conserved GGXG motif of FliM_M_ is involved in the interaction with FliG_M_ (Fig. 2C) [18,30]. FliM_C_ shows significant sequence and structural similarities with FliN and is responsible for the interaction with FliN (Fig. 2D) [35].

### 2.3 FliN

FliN is composed of an intrinsically disordered N-terminal region (FliN_N_) and a compactly folded domain (FliN_C_), which structurally looks similar to FliM_C_ [36]. FliN exists as a dimer of dimer in solution (Fig. 2E) [37] and forms the FliM_1_/FliN_3_ complex along with FliM through an interaction between FliM_C_ and FliN [17]. CheY-P binds to FliN_C_ in a FliM-dependent manner [38]. Leu-68, Ala-93, Val-113 and Asp-116 of *E. coli* FliN are responsible for the interaction with CheY-P (Fig. 2D) [38,39]. The binding of CheY-P to FliN affects interactions between FliM_C_ and FliN, inducing the conformational change of the C ring responsible for directional switching of flagellar motor rotation [38]. FliN_N_ seems to control the binding affinity of FliN_C_ for CheY-P [38] although it is dispensable for the function of FliN [40]. FliN also provides binding sites for FliH, a cytoplasmic component of the flagellar type III protein export apparatus for efficient flagellar protein export and assembly [36,[39], [40], [41], [42]]. Val-111, Val-112 and Val-113 of FliN are responsible for the interaction with FliH (Fig. 2D) [39,41].

### 2.4 Subunit Organization in the C Ring Structure

Electron cryomicroscopy (cryoEM) image analysis has shown that the C ring structures of the purified CCW and CW motors have rotational symmetry varying from 32-fold to 35-fold, and the diameter varies accordingly [43,44]. The C ring diameters of the CCW and CW motors with C34 symmetry are 416 Å and 407 Å, respectively, and so the unit repeat distance along the circumference of the C ring is closer in the CW motor than in the CCW motor [45]. The C ring produced by a *Salmonella fliF–fliG* deletion fusion strain missing FliF_C_ and FliG_N_ lacks the inner lobe, suggesting that FliF_C_ and FliG_N_ together form the inner lobe (Fig. 1) [45,46]. In agreement with this, cryoEM images of the C ring containing the N-terminally green fluorescent protein (GFP) tagged FliG protein show an extra density corresponding to the GFP probe near the inner lobe [47]. The *fliF–fliG* deletion fusion results in unusual switching behavior of the flagellar motor, suggesting that the inner lobe is required for efficient and robust switching in the direction of flagellar motor rotation in response to changes in the environment [45]. The upper part of the C ring wall is formed by FliG_M_ and FliG_C_. FliG_M_ binds to FliG_CN_ of its adjacent FliG subunit to produce a domain-swap polymer of FliG to form a ring in both CCW and CW motors [26,27,29]. Since Helix_Torque_ of FliG_CC_ interacts with MotA_C_ [8,10], FliG_CC_ is located at the top of the C ring wall (Fig. 1). Since FliM_M_ directly binds to FliG_M_ (Fig. 2C) [[18], [19], [20]], the continuous wall of the C ring with a thickness of 4.0 nm and a height of 6.0 nm is formed by side-by-side associations of the FliM_M_ domains (Fig. 1) [32]. A continuous spiral density with a diameter of 7.0 nm along the circumference at the bottom edge of the C ring is made of FliM_C_ and FliN (Fig. 1) [17,36].

## 3 Structural Basis for the Rotational Switching Mechanism

**Fig. 3:**
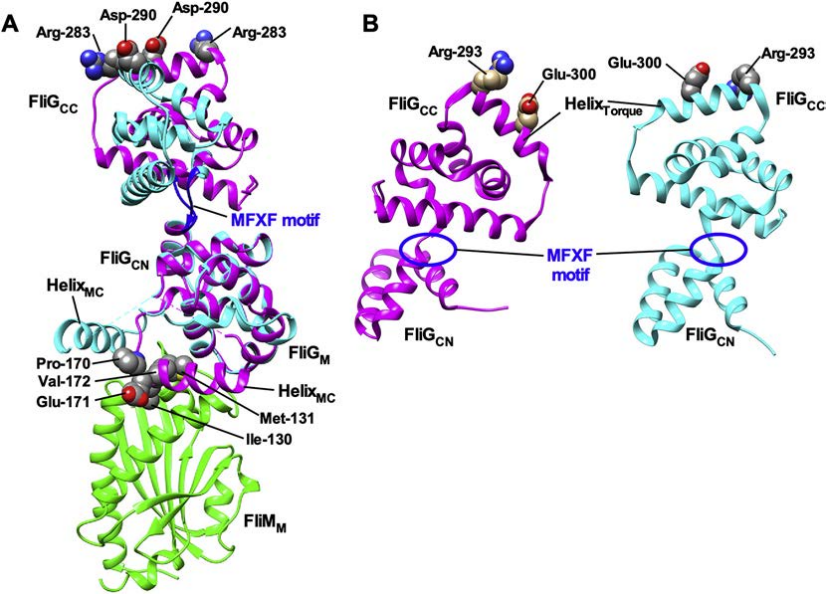
Structural basis for the switching mechanism. (A) Structural comparisons between wild-type FliG_M_ and FliG_C_ domains of *T. maritima* (Tm-FliG_MC_) and its CW-locked deletion variant, Tm-FliG_MC_(∆PEV). Cα ribbon drawing of Tm-FliG_MC_ (magenta), Tm-FliG_MC_(∆PEV) (cyan) and Tm-FliM_M_ (green). The FliG_M_ domain of Tm*-*FliG_MC_(∆PEV) (PDB ID: 3AJC) was superimposed onto that of the Tm-FliG_MC_/Tm-FliM_M_ complex (PDB ID: 4FHR). Helix_MC_ is located at an interface between FliG_M_ and FliM_M_. In contrast, the CW-locked deletion not only induces a distinct orientation of Helix_MC_ relative to the FliG_M_–FliM_M_ interface but also goes through a 90° rotation of FliG_CC_ through a conserved MFXF motif colored in blue. Arg-283 and Asp-290 of Tm-FliG correspond to Arg-281 and Asp-289 of *E. coli* FliG, respectively. (B) Comparisons between the 3USY (cyan) and 3USW (magenta) structures of *Helicobacter pylori* FliG. Conformational rearrangements of the conserved MFXF motif induces a 180° rotation of FliG_CC_ relative to FliG_CN_ to reorient Arg-293 and Glu-300 residues, which correspond to Arg-281 and Asp-289 of *E. coli* FliG, respectively. (For interpretation of the references to colour in this figure legend, the reader is referred to the web version of this article.)

In *E. coli* and *Salmonella*, the flagellar motor is placed in a default CCW state [3,5]. Mutations located in and around Helix_MC_ of FliG, which connects FliG_M_ and FliG_CN_, cause unusual switching behavior of the flagellar motor [48], suggesting that helix_MC_ is involved in switching the direction of flagellar motor rotation. Helix_MC_ is located at the FliG_M_–FliM_M_ interface and contributes to hydrophobic interactions between FliG_M_ and FliM_M_ (Fig. 3A) [18,19]. In-frame deletion of three residues, Pro-Ala-Ala at positions 169 to 171 of *Salmonella* FliG, which are located in Helix_MC_, locks the motor in the CW state even in the absence of CheY-P (CW-locked deletion) [49,50]. The crystal structure of the FliG_M_ and FliG_C_ domains derived from *Thermotaoga maritima* (Tm-FliG_MC_) with this CW-locked deletion have shown that the conformation of Helix_MC_ is distinct from that of the wild-type [19,50,51]. In the wild-type Tm-FliG_MC_/Tm-FliM_M_ complex, Val-172 of Helix_MC_ of Tm-FliG_MC_ makes hydrophobic contact with Ile-130 and Met-131 of Tm-FliM_M_ (Fig. 3A) [18,19]. In contrast, disulfate crosslinking experiments have shown that Helix_MC_ is dissociated from Tm-FliG_M_ in the presence of the CW-locked deletion (Fig. 3A) [28]. Consistently, the CW-locked deletion of Tm-FliG reduces the binding affinity of Tm-FliG_MC_ for Tm-FliM_M_ by about 400-fold [28]. Therefore, it seems likely that the binding of CheY-P to FliM and FliN induces conformational rearrangements of the FliG_M_–FliM_M_ interface, thereby causing dissociation of Helix_MC_ from the interface to facilitate the remodeling of the FliG ring structure responsible for directional switching of the flagellar motor.

Helix_MC_ interacts with Helix_NM_ connecting FliG_N_ and FliG_M_ (Fig. 2A) [23]. The E95D, D96V/Y, T103S, G106A/C and E108K substitutions in Helix_NM_ of *Salmonella* FliG result in a strong CW switch bias [52]. A homology model of *Salmonella* FliG built based on the crystal structure of FliG derived from *A. aeolicus* (PDB code: 3HJL) has suggested that Thr-103 of Helix_NM_ may make hydrophobic contacts with Pro-169 and Ala-173 of Helix_MC_ [45]. These observations lead to a plausible hypothesis that a change in the Helix_NM_–Helix_MC_ interaction mode may be required for conformational rearrangements of the C ring responsible for directional switching of the flagellar motor. A FliF–FliG full length fusion results in a strong CW switch bias of the *E. coli* flagellar motor [27]. Intragenic suppressor mutations, which improve the chemotactic behavior of the *E. coli fliF–fliG* full-length fusion strain, are located at the FliG_N_–FliG_N_ interface [27], suggesting that a change in inter-molecular FliG_N_–FliG_N_ interactions may be required for flagellar motor switching. Therefore, there is the possibility that conformational rearrangements of the FliG_M_–FliM_M_ interface caused by the binding of CheY-P to the C ring influence the Helix_NM_–Helix_MC_ interaction, thereby inducing conformational rearrangements of FliG_N_ domains responsible for the switching in the direction of flagellar motor rotation.

The elementary process of torque generation by stator-rotor interactions is symmetric in CCW and CW rotation [12]. A hinge connecting FliG_CN_ and FliG_CC_ has a highly flexible nature at the conserved MFXF motif, allowing FliG_CC_ to rotate 180° relative to FliG_CN_ to reorient Arg-281 and Asp-289 residues in Helix_Torque_ to achieve a symmetric elementary process of torque generation in both CCW and CW rotation (Fig. 3B) [[53], [54], [55], [56]]. Structural comparisons between Tm-FliG_MC_ of the wild-type and Tm-FliG_MC_ with the CW-locked deletion have shown that the CW-locked deletion induces a 90° rotation of FliG_CC_ relative to FliG_CN_ through the MFXF motif (Fig. 3A) [50]. Consistently, the binding of CheY-P to the C ring induces a tilting movement of FliM_M_, resulting in the rotation of FliG_CC_ relative to FliG_CN_ [34]. Therefore, it is possible that such a tilting movement of FliM_M_ may promote a detachment of Helix_MC_ from the FliG_M_–FliM_M_ interface, resulting in the 180° rotation of FliG_CC_ relative to FliG_CN_.

## 4 Adaptive remodeling of the C ring

**Fig. 4:**
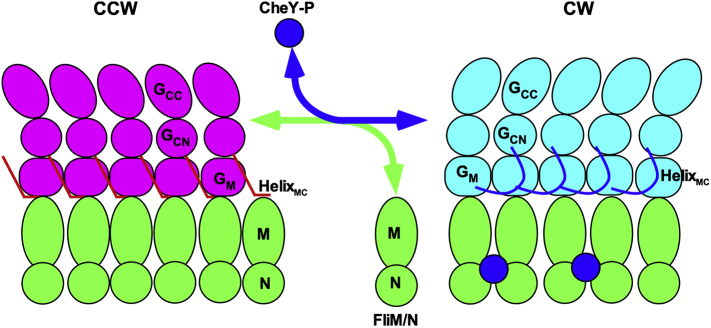
Adaptive remodeling of the FliG ring in the CCW and CW motors. Inter-molecular interactions of FliG_CN_ with FliG_M_ of its neighboring subunit produce the CCW ring structure. Upon binding of CheY-P to the C ring, conformational rearrangements of the FliG_M_–FliG_C_ interface occur, resulting in detachment of Helix_MC_ from the interface. As a result, several weakly bound FliM_1_/FliN_3_ complexes dissociate from the FliG ring.

FliM and FliN alternate their forms between localized and freely diffusing ones (Fig. 4), and the copy number of FliM and FliN in the CCW motor has been found to be about 1.3 times larger than that in the CW motor [[57], [58], [59], [60]]. Consistently, fluorescence anisotropy techniques have shown that the CCW motor accommodate more FliM_1_/FliN_3_ complexes without changing the spacing between FliM subunits [61]. Such exchanges depend on the direction of flagellar rotation but not on the binding of CheY-P to the C ring per se [58]. The timescale of this adaptive switch remodeling of the C ring structure is much slower (~ 1 min) than that of the rotational switching between the CCW and CW states (less than millisecond). Such a structural remodeling of the C ring is important for fine-tuning the chemotactic response to temporal changes in the environments [[62], [63], [64], [65]]. The CW-locked deletion of FliG considerably reduces the binding affinity of FliG_M_ for FliM_M_ presumably due to detachment of Helix_MC_ from the FliG_M_–FliM_M_ interface (Fig. 3A) [28]. Because FliM binds to Helix_MC_ of FliG in the *E. coli* CCW motor [27], the dissociation of Helix_MC_ from the FliG_M_–FliM_M_ interface may promote the dissociation of several weakly bound FliM_1_/FliN_3_ complexes from the FliG ring when CheY-P binds to the C ring to switch from its CCW to CW states (Fig. 4).

## 5 Summary and Perspectives

Switching between the CW and CCW states of the flagellar motor is highly cooperative [66]. The cooperative switching mechanism can be explained by a conformational spread model, in which a switching event is mediated by conformational changes in a ring of subunits that spread from subunit to subunit via their interactions along the ring [[67], [68], [69]]. The binding of CheY-P to FliM and FliN affects subunit-subunit interactions between FliM_M_ domains and between FliM_C_ and FliN in the C ring to induce a 180° rotation of FliG_CC_ relative to MotA_C_, thereby allowing the motor to rotate in CW direction [34]. Helix_MC_ of FliG located at an interface between FliG_M_ and FliM_M_ plays an important role in highly cooperative remodeling of the FliG ring structure [28]. However, it remains unknown how Helix_MC_ coordinates cooperative rearrangements of FliG subunits with changes in the direction of flagellar motor rotation. The C ring of the CCW motor can accommodate more FliM/FliN_3_ complexes without changing inter-subunit spacing, and directional switching of the motor induces several weakly bound FliM/FliN_3_ complexes from the C ring [[57], [58], [59], [60]]. Consistently, the CW-locked deletion weakens an interaction between FliG_M_ and FliM_M_ [28]. Because there is no difference in the rotational symmetry of the C ring between the purified CCW and CW motors [45], it remains unclear how several FliM_1_/FliN_3_ complexes weakly associate with the C ring when the motor spins in the CCW direction.

The elementary process of the torque-generation cycle is symmetrical in CCW and CW directions [12]. However, the output characteristics of the CW motor are distinct from those of the CCW motor. Torque produced by the CCW motor remains almost constant in a high-load, low-load regime of the torque-speed curve and decreases sharply to zero in a low-load, high-speed regime. In contrast, torque produced by the CW motor linearly decreases with increasing motor speed [70]. This suggests that directional switching of the flagellar motor may affect stator–rotor interactions in a load-dependent manner. However, nothing is known about the molecular mechanism. Furthermore, the switching rate of the flagellar motor also depends on the motor speed [71,72]. A recent non-equilibrium model of the flagellar motor switching has predicted that the motor sensitivity to CheY-P increases with an increase in motor torque [73]. However, it remains unknown how stator–rotor interactions modulate the binding affinity for CheY-P. High-resolution structural analysis of the C rings in the CCW and CW states by cryoEM image analysis will be essential to advance our mechanistic understanding of the directional switching mechanism of the flagellar motor.

## Declaration of Competing Interest

The authors declare that they have no competing interests.
